# Using an integrated model of the theory of planned behavior and the temporal self-regulation theory to explain physical activity in patients with coronary heart disease

**DOI:** 10.3389/fpsyg.2023.1049358

**Published:** 2023-02-15

**Authors:** Wenqin Wang, Minjuan Wu, Yun Hua, Xingwei Zhang, Guohe Feng

**Affiliations:** ^1^School of Nursing, Hangzhou Normal University, Hangzhou, Zhejiang Province, China; ^2^School of Public Health, Hangzhou Normal University, Hangzhou, Zhejiang Province, China; ^3^The Affiliated Hospital of Hangzhou Normal University, Hangzhou, Zhejiang Province, China

**Keywords:** physical activity, coronary heart disease, theory of planned behavior, temporal self-regulation theory, consideration of future consequences, habit, self-control

## Abstract

**Background:**

This study aimed to explore the psychosocial determinants of the physical activity (PA) levels in patients with coronary heart disease (CHD) using an integrated theoretical model based on the theory of planned behavior (TPB) and the temporal self-regulation theory (TST).

**Method:**

This was a prospective study conducted at the Affiliated Hospital of Hangzhou Normal University, Zhejiang, China. A total of 279 patients with CHD [176 men aged 26–89 years, mean (M) = 64.69, standard deviation (SD) = 13.17] were selected under the study inclusion criteria by convenience sampling. The data on attitude, subjective norm (SN), perceived behavioral control (PBC), and intention variables for the TPB model and consideration of future consequences (CFC), habit, and self-control (SC) variables for the TST model were collected 1–2 days before the discharge (Time 1, T1) of the participants, and a telephone follow-up was made to assess the participants' self-reported PA levels 1 week after their discharge (Time 2, T2).

**Results:**

The results revealed that only 39.8% of the patients with CHD met the guidelines' recommendations on PA. The data analyses using structural equation modeling (SEM) in the Mplus 8.3 modeling program showed that, in the simple mediation model, attitude, PBC, and CFC were positively related to the intention to practice guideline-recommended levels of PA but SN was not. In addition, intention was shown to mediate the relationships between attitude, PBC, CFC, and PA levels. Furthermore, based on the moderated mediating model, intention and habit were shown to be positively associated with PA levels but SC was not. Moreover, SC played a significant moderating role between intention and PA levels. However, habit strength did not moderate the relationship between intention and PA levels.

**Conclusion:**

An integration of the TPB and TST models offers a good theoretical tool for understanding PA levels in patients with CHD.

## Introduction

Coronary heart disease (CHD) is one of the leading causes of death in China. Approximately 11 million Chinese were diagnosed with CHD in 2017 (Zhou et al., 2019). Physical inactivity accounts for about 6% of CHD cases and contributes to an estimated 0.68-year loss in life expectancy. Therefore, regular physical activity (PA) can potentially prevent the occurrence and recurrence of CHD (Varghese et al., 2016). According to the guidelines discussed by Montalescot et al. (2013) and Arnett et al. (2019) in their studies, achieving about 150 min per week of moderate PA or 60–75 min per week of vigorous exercise is the key to the secondary prevention of CHD. However, PA is a difficult-to-do health-related behavior to establish and maintain. According to various data from 358 surveys, 27.5% of adults globally do not adhere to the health recommendations for PA (Guthold et al., 2018). Moreover, patients diagnosed with CHD tend to engage less in PA than those without a diagnosis of CHD. A previous cross-sectional study reported that about 60% of the enrolled 1,182 patients with CHD in China did not achieve the guideline-recommended levels of PA (Wang et al., 2019). Further, a follow-up study revealed that only 40% of patients with CHD maintained regular PA 12 months after hospital discharge (Wang et al., 2020). Given the health benefits and cost savings associated with regular PA, there is a need to understand the psychological and social factors that affect PA behavior and to develop interventions for promoting adherence to the guideline-recommended levels of PA. Evidence-based theories can help to explain health-related behaviors and develop effective interventions.

The theory of planned behavior (TPB) model is one of the most commonly used models for predicting health-related behaviors. It states that observing health-related behaviors is a reasonable decision-making process (Ajzen, 1991) and suggests that intention determines behavior. Attitude, subjective norm (SN), and perceived behavioral control (PBC) have been reported to positively influence behavior through intention (Ajzen, 1991). Attitude is a comprehensive assessment of behavior based on beliefs about the behavior (views about the benefits, drawbacks, and outcomes of the behavior). A positive attitude about behavior is associated with an increased intention to engage in that behavior. However, SN describes the perceived social pressure from significant others to participate in a particular behavior and is based on one's normative views and one's assumptions about whether or not their close persons would support the behavior. According to the TPB, individuals who feel stronger positive pressure from significant others have an enhanced intention to participate in a particular behavior. Further, PBC is a measure of how a person sees the opportunities, obstacles, and resources needed to engage in a behavior. A higher PBC is associated with a greater intention to engage in a behavior. Notably, the intention to participate in a behavior is mainly determined by attitude, SN, and PBC, and if the intention is strong enough, it will result in a real behavior (Ajzen, 1991).

Intentions cannot fully explain the implementation of behaviors, especially non-hedonistic behaviors (McEachan et al., 2011). The TPB model has been used to explain PA levels in the elderly (Stolte et al., 2017) and patients with diabetes (Plotnikoff et al., 2010) or physical disabilities (Sur et al., 2022). However, the TPB model has not been used to explain PA levels in patients with CHD. According to Elske et al., TPB accounted for 54–60% of the variance in intention to PA (Stolte et al., 2017). However, it only accounted for 13–16% of the variance in PA behavior. Moreover, Plotnikoff et al. reported that the TPB model accounted for ≥40% of the variation in intentions for types 1 and 2 diabetes. However, the TPB only explained 23 and 19% of the variance in actual PA behavior for types 1 and 2 diabetes, respectively (Plotnikoff et al., 2010). A meta-analysis investigating the ability of the TPB to evaluate for PA among individuals with physical disabilities revealed that intention had a moderate effect on PA, attitude had a moderate effect on intention, SN had an insignificant effect on intention, and PBC had a moderate effect on intention and a non-significant direct effect on PA (Sur et al., 2022). Furthermore, Hagger et al. revealed that the TBP model could explain a 43.7–49.6% variance of intention to PA behavior among the general population but only a 21.2–22.2% variance of actual PA behavior (Hagger et al., 2002). Overall, these studies indicated that the TPB model can effectively explain the intention to engage in PA behavior. However, there was still a large amount of variance between intention to behavior and PA behavior, demonstrating that intention may not be the only variable influencing health-related behaviors. Intention–behavior gap refers to the existence of discrepancies between intention and actual behavior, and it suggests the existence of other variables that could affect behavior.

The temporal self-regulation theory (TST) is a dual-process theory (Hall and Fong, 2007), which proposes that automatic (behavioral prepotency) and rational (intention and self-regulatory capacity) processes directly affect behavior. In the motivational stage, this theory proposes that connectedness beliefs and time perception are the key determinants for understanding future-oriented health-related behavior and can predict behavioral intention. Connectedness beliefs refer to how people think their current actions affect future outcomes. Time perception is a measure of the degree to which one thinks about the future consequences of present behaviors (Hall and Fong, 2007). It is conceptualized as “consideration of future consequences (CFC)” (Strathman et al., 1994). To achieve the guideline-recommended levels of PA, individuals have to sacrifice immediate pleasure for long-term health benefits (Gellert et al., 2012). Furthermore, people should focus on the long-term consequences and should be motivated to act in the present to achieve the guideline-recommended levels of PA (Villaron et al., 2017). Consequently, we postulate that time perception promotes rational intention to achieve the guideline-recommended levels of PA for health benefits.

To narrow the “intention–behavior” gap, the TST proposes that behavioral prepotency and self-regulatory capacity can directly influence actual behavior or moderate the association between intentions and behaviors (Hall and Fong, 2007). Behavioral prepotency involves the likelihood of engaging in a behavior given the past performance frequency, the degree to which the behavior is habitual, and the situational demands around that behavior, such as contextual cues to action (Hall and Fong, 2007). According to previous research, habit strength, environmental cues, and past behavior were significantly related (Black et al., 2017). Therefore, similar to earlier research, we may appropriately represent behavioral prepotency with habit strength in this study (Allom et al., 2013). Habit strength refers to developing a learned reaction to a cue (Gardner and Lally, 2018) and is an unconscious response that might influence behavior (Baumeister R. F. et al., 2007; Hall and Fong, 2007). Allom et al. demonstrated the importance of habit strength in maintaining long-term behaviors such as engaging in PA (Allom et al., 2018). For a behavior to become habitual, the intention of the behavior must first be established. Further, as the behavior is repeated, it may become routine and driven by unconscious processes (Hall and Fong, 2007; Wood and Rünger, 2016). Therefore, habit strength may act as a moderating variable in the association between intention and PA behaviors, such that intentions can only predict actual behavior among people with weak habits (Gardner, 2015; Rebar et al., 2016).

Achieving goal-oriented behaviors requires motivation and the capacity to act on the drive. Self-regulation capacity refers to the cognitive capacity to keep one's thoughts, emotions, and behaviors under control (Baumeister R. et al., 2007). According to Hall et al., individuals with stronger self-regulatory capacity are more likely to behave in tandem with their intentions (Hall et al., 2008). Self-control (SC) is a vital feature of self-regulatory capacity and is a significant factor that determines health-related behavior (Tangney et al., 2004). A previous cross-sectional study assessing the intention–behavior gap by measuring the difference between the participants' subjective PA standard and their actual PA behavior revealed that the participants with greater SC had a smaller “intention–behavior” gap than those with less SC (Bertrams and Englert, 2013). This suggests that individuals with higher SC may have higher levels of PA and fewer discrepancies between intentions and actual behaviors (Hagger et al., 2010; Englert, 2016).

**Figure 1:**
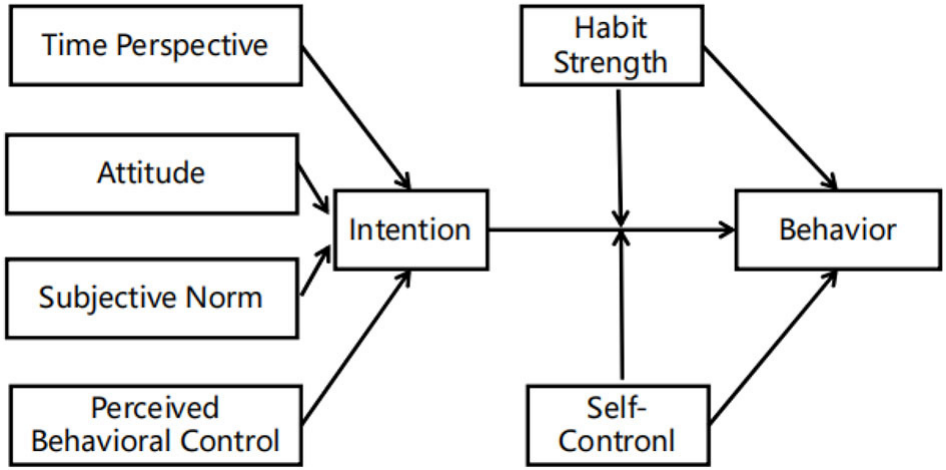
An integrated model of the theory of planned behavior (TPB) and the temporal self-regulation theory (TST).

The two theoretical models of health-related behavior, TPB and TST, have several strengths. The connectedness belief of the TST model is comparable to the attitude of the TPB model. However, in previous studies, the connectedness belief only accounted for a small variance in intention (Black et al., 2017; Evans et al., 2017; Jones and Schüz, 2022). Several meta-analyses exploring the pre-intention variables of the TPB model predicted a moderate-to-large variance in intention to PA behavior on average (Hagger et al., 2002; Downs and Hausenblas, 2005; McEachan et al., 2011). Furthermore, the previous studies integrating the two theories accounted for a greater variance in health-related behavior than using the TPB alone (Hagger et al., 2002; Liddelow et al., 2021a,b; Mullan et al., 2021). Additional variables can be added to the TPB model framework to boost its explanatory power, which is a major strength of this model (Ajzen, 1985). Therefore, this study aimed to analyze the explanatory power of integrating the pre-intentional variables of the TPB model and the volitional stage of the TST model on health-related PA behavior in patients with CHD (see Figure 1).

Several studies examined the predictive efficiency of the constructs of the TPB and TST models in PA behavior. However, patients with a diagnosis of CHD are faced with distinct physical and psychological barriers, which restrict them from participating in moderate-to-vigorous physical activity (MVPA). For instance, patients who experience angina during exercise or rest may develop a fear of engaging in PA (Deka et al., 2021). Therefore, they may engage in lower-intensity PA and show reduced adherence to PA. This calls for studies to understand the factors that underlie PA behavior change from the CHD context. This study aimed to investigate the rate of compliance with PA in patients with CHD according to the ACC/AHA guidelines. In addition, this study aimed to explore the determinants of practicing a guidelines-recommended level of PA using a combination of the TPB and TST models (see Figure 1) in a specific clinical setting with patients with CHD. Demographics and clinical characteristics, such as age, gender, New York Heart Association (NYHA) Classification of cardiac function, and number of comorbidities (Stewart et al., 2013; Alkerwi et al., 2015; Peersen et al., 2020) were collected as controlled variables because they were known to significantly affect PA behavior. We theorized that CFC may be beneficial to supply the additional variance of PA's intention and that the relevant variables of habit strength and SC may assist in closing the gap between intention and actual PA behaviors.

## Aims and hypothesis

This prospective study aimed to investigate the effect of CFC and the TPB variables (attitude, SN, and PBC) on the intention of practicing recommended levels of PA. Then, we aimed to analyze how habit and SC of the TST can be used to explain PA levels. The hypotheses of this study are as follows:

**H1:** Consideration of future consequences and variables of the TPB model (attitude, SN, and PBC) are positively associated with the intention to practice guidelines-recommended levels of PA in patients with CHD.
**H2:** Variables of TST (intention, habit strength, and SC) are positively associated with the levels of PA behavior.
**H3:** Intention to practice guidelines-recommended level of PA mediates the relationships between CFC, attitude, SN, PBC, and PA levels.
**H4:** Habit strength of PA plays a significant moderating role between intention and PA levels, specifically, in patients with higher habit strength, the intention was not significantly correlated with PA levels.
**H5:** Self-control plays a significant moderating role between intention and PA levels, specifically, in patients at higher levels of SC, the relationship between intention and PA levels will be stronger.

## Materials and methods

### Study design and participants

A prospective research design with two time points for measurement was utilized in this study. Approval to conduct the study was sought from the Ethical Committee of the Affiliated Hospital of Hangzhou Normal University, Zhejiang, China (Approval No.: 2022(E2)-HS-060). A total of 279 patients with CHD treated at the Hangzhou Normal University, Zhejiang, China from February to July 2022 were included through a convenience sampling method. The inclusion criteria were as follows: (1) patients aged ≥ 18 years; (2) patients meeting the diagnostic criteria for stable CHD as per the Guidelines for the Diagnosis and Treatment of Stable Coronary Heart Disease in China (2018) (Editorial Board of Chinese Journal of Cardiology, 2018); (3) the time for meeting the diagnostic criteria was ≥3 months; (4) patients with NYHA class I or II symptoms; and (5) signed informed consent. The exclusion criteria were as follows: (1) patients with severe complications or other organic failure limiting their PA behavior, including myocardial infarction, heart failure, and congenital heart disease; (2) patients with mental illnesses; (3) patients with hearing impairment or communication disorders; and (4) pregnant patients.

This study comprised experiments performed at two time points. Before completing measurements, all participants were asked to sign an informed consent form and give their telephone number through which they can be contacted for the second part of this study. A paper-and-pencil survey was administered to the participants by a well-trained research assistant 1–2 days before discharge (Time 1). The survey lasted about 15 min. The study participants were provided with a manual detailing information on self-management for CHD to assist them in locating support for their particular requirements. To ensure a 100% follow-up rate at Time 2, we asked the participants what time of day was convenient to answer the phone and informed them that they would receive a 10-min telephone follow-up assessment of PA behavior 1 week after their discharge. Finally, we connected their responses from Time 1 and Time 2. The participants did not receive any monetary compensation for participating in this study.

### Measurements

#### Demographics and clinical characteristics

All participants provided self-reports on their demographics, including gender, age, and level of education. The clinical characteristics, such as NYHA classification of cardiac function, and several comorbidities were reported by their attending doctors. However, the duration of the disease was self-reported by the participants. Further, the weight and height of the patients were measured and used to calculate the body mass index (BMI) based on the following formula: BMI = weight (kg) ÷ [height (m)]^2^ (Obesity and World Health, 2000).

#### Consideration of future consequences scale

Time perception was evaluated using the Chinese version of the 12-item CFCS (Feng et al., 2020). It was originally developed by Strathman et al. (1994). The scale has a scoring range from 1 (extremely uncharacteristic) to 7 (extremely characteristic). The CFCS measures how much people think about the future when making decisions. It has two subscales considering the immediate (reverse coded) and future dimensions. A higher score indicates a greater future time perception. The original version showed good psychometric properties (Cronbach's α: CFC-I = 0.84; CFC-F = 0.80) (Strathman et al., 1994). Further, the Chinese version also showed good reliability (Cronbach's α = 0.71–0.91) (Feng et al., 2020). The CFSC for this study also showed good reliability (Cronbach's α: CFC-I = 0.786; CFC-F = 0.727; and CFCS = 0.865).

#### Variables of TPB

The participants were informed of the recommended levels of weekly PA behavior as per the American College of Cardiology/American Heart Association (ACC/AHA) guidelines (Arnett et al., 2019). Furthermore, all participants completed a Chinese version of the TPB questionnaire (Hu and Ma, 2008), which was originally developed and validated by Ajzen (1991) with good reliability. The average scores on each subscale were calculated.

The attitude was evaluated using a 5-item scale by completing the phrase, “For me, performing the recommended level of PA during the next week would be …”. The patients were supposed to choose an answer from each of the following items: Enjoyable–unenjoyable; pleasant–unpleasant; satisfied–unsatisfied; useful–useless; important–unimportant. The total scores of each participant were averaged with a higher score indicating a more positive attitude. This subscale had a Cronbach's α of 0.934.

Subjective norm was assessed based on 3 items using responses on a 6-point Likert scale ranging from 1 for “strongly disagree” to 6 for “strongly agree.” The participants were required to respond to “the majority of people important to me expect me to do the recommend-level of PA during the next week.” A higher mean score indicated a more positive perceived SN for PA. This subscale had a Cronbach's α of 0.885.

Perceived behavioral control was assessed by three items using responses on a 6-point Likert scale ranging from 1 for “strongly disagree” to 6 for “strongly agree.” The participants were asked to respond “if I wanted to, I could do the recommended level of PA during the next week.” A higher mean score indicated a higher PBC. This subscale had a Cronbach's α of 0.919.

The intention was assessed by three items using responses on a 6-point Likert scale ranging from 1 for “strongly disagree” to 6 for “strongly agree.” The participants were asked to respond to “I will attempt to do the recommended level of PA during the next week.” A higher mean score indicated a greater intention. This subscale showed excellent reliability with a Cronbach's α of 0.944.

#### Variables of temporal self-regulation theory

The Self-Report Habit Index (SRHI) (Verplanken and Orbell, 2003) was used to measure the habit strength of practicing the guideline-recommended level of PA. This scale demonstrated good psychometric properties in Chinese teenagers (Chu and Xiao, 2020). The SRHI consists of a stem “Behavior X… is something …” that is adapted for different behaviors (e.g., “practicing guidelines-recommended level of physical activity is something …”), followed by 12 items with 7-point Likert scale responses ranging from 1 for “completely disagree” to 7 for “completely agree.” The items were averaged to obtain a total SRHI score ranging between 1 and 7. This index showed good reliability with a Cronbach's α of 0.938.

Self-control was assessed by the Chinese version of the Brief Self-Control Scale (BSCS) (Luo et al., 2021), which was originally developed by Morean et al. (2014). The BSCS is a reliable measurement of self-discipline and impulse control, comprising 7 items measured on a 5-point Likert scale. The participants were asked to choose the answer that best reflected them, with higher scores indicating greater SC. The Chinese version of BSCS showed a Cronbach's α of 0.83 (Luo et al., 2021). The BSCS in this study showed good reliability (Cronbach's α = 0.859).

#### International physical activity questionnaire-long form (IPAQ-long form)

The Chinese version of the IPAQ-long form was used to assess PA behavior for the last 7 days, including frequency and duration of time spent in the following four domains: Occupation, transportation, housework, and recreational PA (Craig et al., 2003; Qu and Li, 2004). Metabolic equivalents (METs) were used to quantify the intensity of PA as low (3.3 METs), moderate (4.0 METs), or high (8.0 METs) (Fan et al., 2014). The following formula was used to calculate the total MET-min/week: low (MET × min × days) + moderate (MET × min × days) + high (MET × min × days) (Fan et al., 2014). According to the ACC/AHA guidelines' recommendations on PA (patients with CHD should engage in at least 150 min of moderate PA or 75 min of vigorous PA weekly or an equal combination of the two) (Montalescot et al., 2013; Arnett et al., 2019). Therefore, the participants were classified into those meeting the ACC/AHA recommendations on PA in patients with CHD or those not meeting the ACC/AHA recommendations on PA in patients with CHD.

### Statistical analysis

To ensure accuracy, the data obtained were recorded using Microsoft Excel 2019 by double entry method and analyzed in Statistical Package for the Social Sciences (SPSS), version 25.0 (IBM, USA) and Mplus 8.3 modeling program. The normality of the model's variables was evaluated using the skewness statistic and the normal probability plot and found that every variable in the combined theory model had a skewed distribution. Similar to the previous studies (Stewart et al., 2013; Alkerwi et al., 2015; Peersen et al., 2020), we used the chi-square test, *t*-test, and analysis of variance (ANOVA) to check for differences in intention to PA and PA levels, such as age (e.g., < 60 or ≥60 years), gender, levels of education, NYHA classification of cardiac function, duration of being diagnosed with CHD (e.g., < 1, 1–5, or ≥5 years), and several comorbidities (e.g., 0, 1, or ≥2), to determine whether or not these factors should be controlled. There was only a significant association in the NYHA classification of cardiac function and intention; hence, this was chosen as the covariate for intention. Age, gender, NYHA classification of cardiac function, and a number of comorbidities were all found to be significantly associated with levels of PA, and therefore, these variables would be controlled to PA levels. Descriptive statistics and Spearman bivariate correlations on the unstandardized variables were performed (see Table 1).

**Table 1:** Means, standard deviations, and bivariate correlations between the variables of the integrated theoretical model (*N* = 279).

**Variable**	**M ± SD**	**1**	**2**	**3**	**4**	**5**	**6**	**7**	**8**	**9**	**10**	**11**	**12**	**13**	**14**
1. Gender	–	–													
2. Age	–	0.237^**^	–												
3. BMI	–	−0.073	−0.192^**^	–											
4.Edu	–	−0.181^**^	−0.307^**^	−0.031	–										
5. Duration of disease	–	0.115	0.305^**^	−0.053	−0.184^**^	–									
6. NYHA	–	0.033	0.090	−0.024	−0.124^*^	0.094	–								
7. Number of comorbidities	–	−0.053	0.225^**^	−0.123^*^	0.020	0.036	0.137^*^	–							
8. Attitude	3.69 ± 1.15	−0.008	0.005	0.026	−0.006	−0.033	−0.087	−0.064	–						
9. SN	4.10 ± 1.27	0.130^*^	0.029	−0.088	0.046	−0.083	−0.215^**^	−0.044	0.465^**^	–					
10. PBC	3.53 ± 1.39	0.006	0.047	–0.064	0.054	−0.007	−0.074	−0.027	0.529^**^	0.465^**^	–				
11. CFC	4.12 ± 0.75	0.051	0.031	−0.055	0.078	−0.012	−0.151^*^	−0.021	0.324^**^	0.275^**^	0.472^**^	–			
12. Intention	3.73 ± 1.53	−0.018	0.025	−0.018	0.095	0.065	−0.145^*^	−0.071	0.565^**^	0.399^**^	0.685^**^	0.559^**^	–		
13. Habit	3.81 ± 0.70	0.040	−0.095	−0.067	−0.008	−0.041	−0.126^*^	−0.088	0.081	0.112	0.209^**^	0.204^**^	0.194^**^	–	
14. SC	3.14 ± 0.70	−0.018	0.074	0.042	−0.116	0.071	0.052	0.059	0.052	0.125^*^	0.217^**^	0.106	0.234^**^	0.077	–
15. PA	–	−0.121^*^	−0.261^**^	−0.109	0.154^*^	−0.058	−0.168^**^	−0.138^*^	0.157^**^	0.123^*^	0.304^**^	0.150^*^	0.338^**^	0.313^**^	0.087

M, mean; SD, standard deviation; Edu, education; CFC, consideration of future consequences; SN, subjective norms; PBC, perceived behavioral control.

Associations between dichotomous and continuous variables are point bi-serial correlations. Gender (men = 0, women = 1), Age (< 60 years = 1, ≥60 years = 2), body mass index (BMI) (BMI < 24 kg/m^2^ = 1, 24 kg/m^2^ ≤ BMI < 28 kg/m^2^ = 2, BMI > 28 kg/m^2^ = 3); Education (only completed the primary school or not = 1, junior high school = 2, senior high school or technical secondary school = 3, a 3-year college or above = 4), NYHA (NYHA class I = 1, NYHA class II = 2); Duration of disease [being diagnosed with stable coronary heart disease (CHD) for < 1 year = 1, being diagnosed more than 1 year, but < 5 years = 2, being diagnosed more than 5 years = 3]; Number of comorbidities (no comorbidities = 0, one comorbidity = 1, at least two comorbidities = 2), PA levels (not meeting the guideline-recommended level = 0, meeting the guideline-recommended level = 1).

^*^p < 0.05; ^**^p < 0.01.

Taking into account the benefits of Mplus 8.3, we conducted structural equation modeling (SEM) analysis in Mplus 8.3 to examine the mediating role of intention as well as the moderating role of habit and SC in the second stage of the mediation. Before the analysis, item parcels were selected to be observed indicators using the item-to-construct balance suggested by Little et al. (2002), and it was employed to measure CFC, habit, and SC. To determine whether or not the mediation effects were statistically significant, the bootstrapping technique was used, with a sample size of 5,000, and generated 95% confidence intervals (CIs) that were adjusted for bias. Subsequently, the latent moderated structural (LMS) equations were employed in the moderated mediation model. The goodness-of-fit model was measured using the comparative fit index (CFI), the Tucker–Lewis index (TLI), and the root mean square error of approximation (RMSEA), each with a cutoff value of >0.90, >0.90, and 0.08, respectively; a *p*-value of < 0.05 was considered statistically significant, and all tests were two-sided ones.

#### Common method bias

Since the data were acquired from a single self-reported source, Harman's single factor test (Podsakoff et al., 2012) was employed to investigate whether there was a common method bias. Nine major components were established (with an eigenvalue >1). Only 23.64% of the total variation could be explained by the first principal component, which is much below the standard value of 40%. Therefore, the common method bias was less likely to influence the results of this study.

## Results

### Study participants

Time 1 included 282 participants. However, data from three participants were excluded from analyses due to too much missing data. Therefore, only 279 participants were included in the final analyses. The participants were aged 26–89 years (*M* = 64.69, *SD* = 13.17) with a mean BMI of 25.01 kg/m^2^ (*SD* = 3.78). Time 2 of this study collected the PA levels of the 279 participants through telephone follow-up. The telephone follow-up rate was 100%.

Most participants (*n* = 168, 60.2%) did not achieve the guideline-recommended levels of PA. However, 39.8% (*n* = 111) of participants achieved the guideline-recommended levels of PA. Also, 43% of the participants were not overweight or obese (BMI < 24 kg/m^2^), 32.6% were identified as overweight (24 kg/m^2^ ≤ BMI < 28 kg/m^2^), and 24.4% were obese (BMI > 28 kg/m^2^). Most participants were male patients (*n* = 176, 63.1%). In addition, 190 (68.1%) participants were elderly (age ≥ 60 years). Most participants (*n* = 112, 40.1%) had only completed primary school or not, 82 (29.4%) had completed junior high school, 53 (19.0%) had completed senior high school or technical secondary school, and 32 (11.5%) had a 3-year college or above. A total of 87.8% of participants (*n* = 245) were in NYHA class I. Additionally, most participants (*n* = 104, 37.3%) had lived with stable CHD for < 1 year, while 31.5% of participants had lived with stable CHD for more than 5 years. Furthermore, 125 participants (44.8%) had one comorbidity, 103 (36.9%) participants had at least two comorbidities, and 51 (18.3%) participants had no comorbidities.

### Measurement model

A preliminary examination of the measurement model was carried out using confirmatory factor analysis before several structural modes were performed. The measurement model comprised seven latent constructs that included CFC, attitude, SN, PBC, intention, habit, and SC. The measurement model exhibited a satisfactory model–data fit, with CFI = 0.995, TLI = 0.994, and SRMR = 0.028. Standardized factor coefficients were obtained within the acceptable range of 0.828–0.847 for CFC; 0.809–0.916 for attitude; 0.820–0.874 for SN; 0.851–0.947 for PBC; 0.891–0.936 for intention; 0.860–0.950 for habit; and 0.718–0.903 for SC, which are all above the acceptable criteria of 0.6.

### Simple mediation model

**Figure 2:**
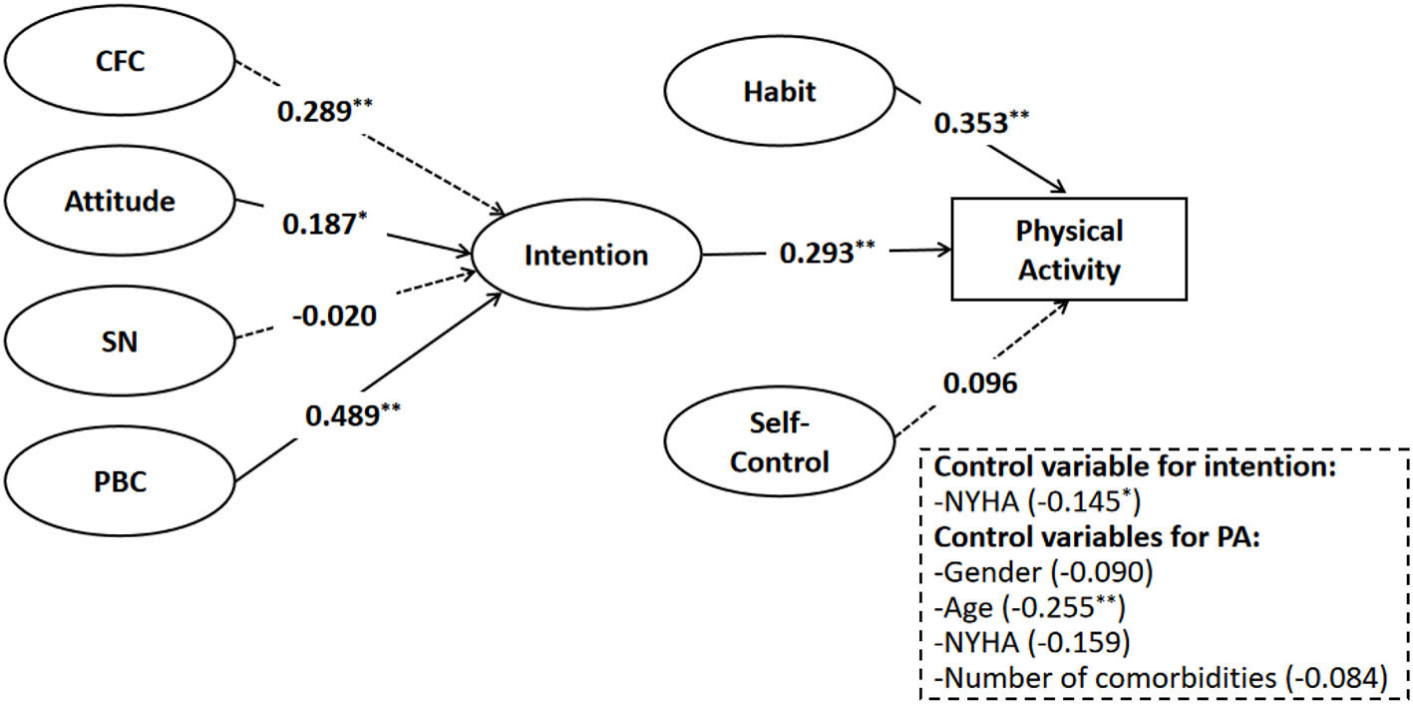
Simple mediation model. *p < 0.05; **p < 0.01.

Two different types of structural models were calculated based on the hierarchical method. Before examining the moderating effect of habit and SC in the link between intention and PA levels, we developed a simple mediation model without examining the moderating medication interactions. As mentioned above, the NYHA variable was controlled for intention, gender, age, and NYHA, and the number of comorbidities was controlled for PA behavior. Since PA behavior is a categorical variable, the PROBIT connection in conjunction together with the weighted least squares mean and variance (WLSMV) adjusted technique was employed to fit the data. The PROBIT link function is efficient in estimating models that include categorical outcomes and can obtain model fit indices. The simple mediation model achieved a good level of fitness with CFI = 0.976; TLI = 0.973; and RMSEA = 0.020. Furthermore, this simple mediation model accounted for 42.8% of the variance in PA levels (see Figure 2).

The findings of the simple mediation model were partially consistent with H1 and H2 (see Figure 2). First, CFC, attitude, and PBC were found to be directly and positively correlated with the intention to practice guideline-recommended levels of PA behavior: *b* = 0.289, standard error (SE) = 0.086, *p* < 0.05; *b* = 0.187, SE = 0.083, *p* < 0.05; *b* = 0.489; SE = 0.151, *p* < 0.05, respectively. However, SN was not associated to achieve the guideline-recommended levels of PA behavior (*b* = −0.020, SE = 0.109, *p* = 0.853). Second, we examined how intention, habit, and SC influenced the PA levels. The results indicated that both intention and habit directly and significantly influenced PA levels (*b* = 0.239; SE = 0.077, *p* < 0.001; *b* = 0.353, SE = 0.069, *p* < 0.001, respectively). Furthermore, SC was not associated with PA levels (*b* = 0.096, SE = 0.078, *p* = 0.220). In addition, we examined the mediating role of intention in the proposed combined theoretical model. The mediation effects were tested using the bootstrapping method. Except for SN, the results from 5,000 iterations indicated that indirect pathways linking CFC, attitude, and PBC to PA levels through intention were all significant (see Table 2). In addition, the mediating effects were significant with no zeros between the lower and upper bounds of the 95% CI. Therefore, H3 was partially supported (see Table 2).

**Table 2:** Results of the mediating effect.

**Mediating effect**	***B***	**SE**	***p***	**BC 95% CI**	

				**Lower**	**Upper**
CFC → Intention → PA	0.085	0.035	0.016	0.027	0.144
Attitude → Intention → PA	0.055	0.031	0.078	0.006	0.111
SN → Intention → PA	−0.006	0.037	0.872	−0.062	0.037
PBC → Intention → PA	0.143	0.068	0.036	0.056	0.269

SN, subjective norms; PBC, perceived behavioral control; CFC, consideration of future consequences; PA, physical activity; BC, biased corrected.

### Moderated mediation model

**Figure 3:**
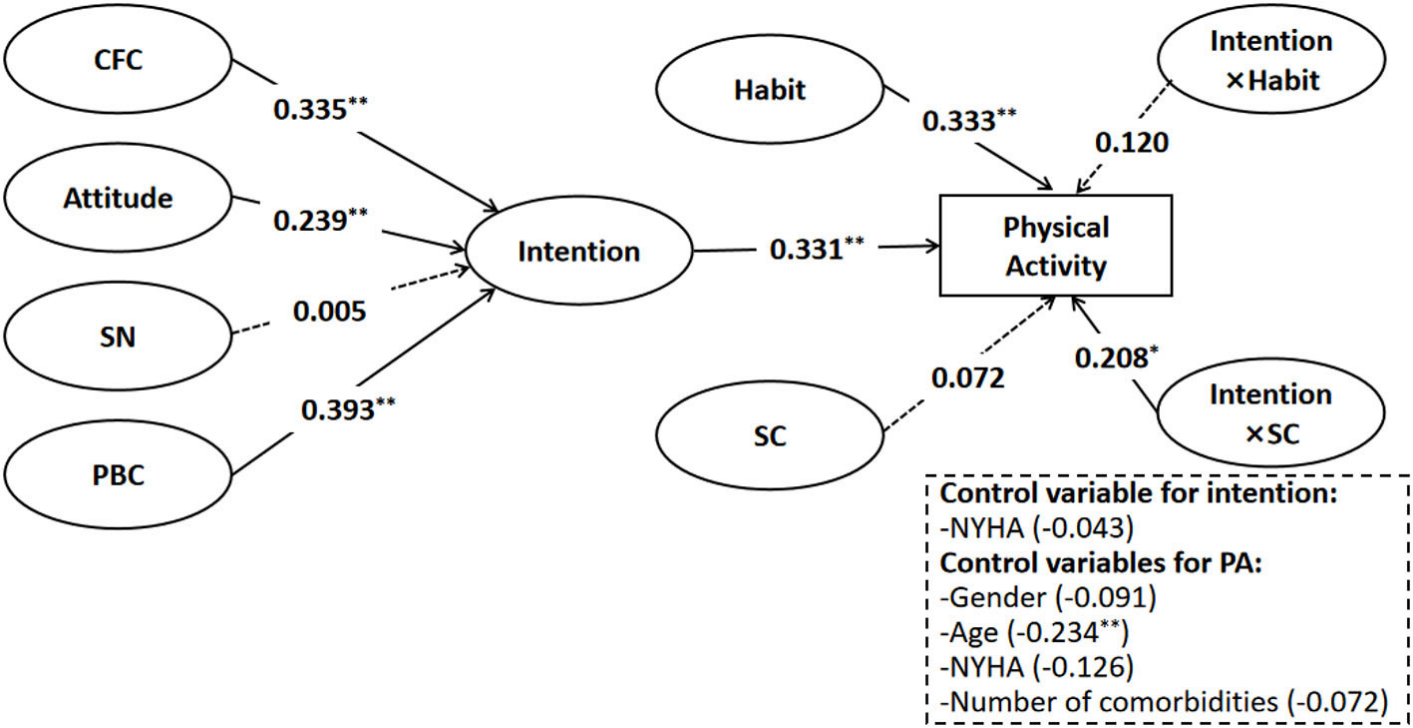
Moderated mediation model. *p < 0.05; **p < 0.01.

Analysis of the simple mediation model revealed that CFC, attitude, and PBC increased the PA levels by increasing the intention to practice the guideline-recommended levels of PA. However, some parts of these casual sequences were dependent on the participants' levels of habit and SC. Thus, two interaction terms (intention × habit and intention × SC) were added to the simple mediation model. The model was developed with the path connecting intention to PA levels varying with a participant's level of habit strength and SC (see Figure 3). Due to the inability of WLSMV to analyze latent variable interactions, maximum likelihood estimation with robust standard errors (MLR) was used together with TYPE = RANDOM and a logit link. Fit statistics were not available in these computational settings.

Figure 3 shows information regarding the estimated relationships, including whether or not a certain path is statistically significant and corresponding standardized coefficients. The results in Figure 3 showed that the interaction term “intention × SC” had a positive impact on PA levels (*b* = 0.208, SE = 0.082, *p* = 0.011, OR = 1.643). However, the term “intention × habit” was not statistically significant. Specifically, patients who showed higher intention to practice PA exhibited a higher level of PA and were more accurate in those with higher SC. This finding suggests that moderation occurs only in terms of the overall mediating process.

The significant paths for controlled variables were consistent with the simple mediation model. Patients with CHD with NYHA I classification showed a higher intention to achieve the AHA/ACC guideline's recommended level of PA. In addition, older patients were less likely to practice the AHA/ACC guideline's recommended level of PA.

## Discussion

In this study, we integrated the TPB (Ajzen, 1991) and TST (Hall and Fong, 2007) models to determine whether the two models can help to close the gap between the intention to PA and actual PA levels in patients with CHD. The results revealed that CFC, attitude, and PBC had significant effects on the intention to achieve the ACC/AHA guidelines' recommended level of PA, partially supporting H1. In addition, SN was not significantly related to intention in the simple mediation model. Second, the results partially supported H2 by revealing that intention and habit strength could significantly explain PA levels. However, SC was not statistically significant in the simple mediation model. Third, H3 was partially supported by the mediating effects of intention between CFC, attitude, PBC, and PA levels. However, there were no mediating effects of intention between SN and PA levels. Fourth, H4 was not supported since habit strength of guideline-recommended PA levels was shown to have no significant moderating effect on the relationship between intention and PA. Finally, H5 was supported by the finding that SC had a significant moderating effect on the association between intention and PA levels, indicating that a positive association between intention and PA behavior was more likely to be obtained at higher levels of SC. Overall, the integrated theoretical model is a useful theoretical tool for explaining PA behaviors in patients with CHD.

## Theoretical implications

The findings of this study have important theoretical implications for the commonly used behavior change theories. Specifically, this study focuses on specific clinical settings and aimed to reveal the psychological mechanisms of PA intentions and PA behaviors. First, our findings are in line with previous studies and the significance of the motivational aspects of PA (Sassen et al., 2010). We demonstrate that the patients are more likely to engage in MVPA if they are made aware of the benefits of PA, and when they have a positive experience engaging in such activity. Second, our results showed that patients with CHD may consider their perceived control of PA to be the most essential factor in determining whether or not they intend to achieve a guidelines-recommended level of PA. This is congruent with the findings from adults with similar clinical characteristics (Johnston et al., 2004). However, our results contradict those from previous literature regarding adults without CHD (Downs and Hausenblas, 2005), which demonstrated that attitude had the highest impact on intention. This discrepancy may imply a distinct mechanism of intention generation in patients with CHD. Patients with CHD may experience feelings of physical constraint, bad mood, and unfavorable perceptions about life following the cardiac incident as the self-perceived barriers that restrict them from engaging in PA (Alharbi et al., 2017; Deka et al., 2021). Third, in the integrated theoretical model, CFC acted as a measure of the temporal framework within which perceived results are assessed. Moreover, results showed that CFC was linked to the intention to achieve the guidelines-recommended level of PA, and indicated that future time orientation may contribute to PA behaviors by enhancing motivation to engage in them. This is consistent with a prior study conducted by Beek et al. (2017) but inconsistent with another study (Hamilton et al., 2003) due to the different measures of time perception. Notably, the majority of patients with CHD were elderly (Länsitie et al., 2022), suggesting that, in contrast to young individuals, they may have a pessimistic outlook on life expectancy, especially when considering present limitations such as pre-existing illnesses (Hamilton et al., 2003). Therefore, healthcare practitioners can explain the likelihood of specific behaviors and behavioral consequences with more accuracy if they account for factors such as CFC.

Further, our results underscore the importance of the automatic process of PA behaviors. Engaging in PA often entails repeating the same activity in the same context (such as having a brisk walk on the way to work), thus these behaviors may develop into a habit over time. Therefore, in these routinely rewarding and predictable settings, automatic or habitual processes predominated (Phillips and Mullan, 2022). Participants with stronger habit strength of practicing a guideline-recommended level of PA were more likely to behave in practice, which is consistent with findings reported previously (Gardner et al., 2011) and the combined theoretical model. However, the effect of habit on moderating the relationship between behavior and intention to achieve the guidelines-recommended level of PA was not investigated in this particular study. This differs from the theoretical model (Hall and Fong, 2007) and the results of previous studies investigating the effect of intention × habit interaction on PA behavior (de Bruijn and Rhodes, 2011; Gardner et al., 2011; van Bree et al., 2013). These results further prove the viewpoint of Gardner (2009) that individual habitual behavior and behavioral intention are two parallel and substitutable variables. That is, before the habit of PA is formed, behavioral intention is the primary determinant of PA levels, but after the habit is formed, it is mainly determined by its habit.

Self-control did not have a direct relationship with actual behavior in the simple mediation model, which contradicts empirical findings from previous investigations (Hagger et al., 2002; de Ridder et al., 2012) and the integrated theoretical model. The measurement of SC was not specific to PA behavior in this study; thus, the ability of SC to explain the variance of PA behavior may be underestimated (Mullan et al., 2021). However, the observed significant variance in PA levels may be explained by the interaction between intention and SC. This implied that a positive link was observed between intention and PA levels at high levels of SC, but when SC was low, there was no significant link. This finding is consistent with theoretical assumptions (Hall and Fong, 2007; Hagger et al., 2010) and prior empirical evidence (Pfeffer and Strobach, 2017), which indicates that intention is not sufficient to carry out behavior, and SC has required the formation of behavior. Consistently engaging in regular PA makes a great demand on one's capacity for self-regulation (Hagger et al., 2016) as it often involves conflicts between instant satisfaction (like relaxing and playing on a phone) and delayed gratification (like accomplishing something important) (e.g., satisfying healthy goal). Thus, individuals with a high level of SC are more possible to practice discipline regardless of the situation or environment, and this might enable them to overcome barriers that prevent the implementation of PA intention into behavior.

Finally, this study extends the application of the constructs of the TPB and TST in clinical settings. The TPB has been widely used in different contexts for different health behaviors, but few studies have explored its application in PA behavior in specific clinical contexts. This study extends the theory to delineate patients' rational and habitual changes in fostering their PA behavior in a temporal framework. The present results are consistent with the proposal that both automatic (behavioral prepotency) and rational (intention and self-regulatory capacity) processes are crucial for understanding, predicting, and modifying health-related behaviors.

## Practical implications

The current investigation also provides health practitioners with important recommendations for promoting PA levels in patients with CHD. Initially, the perceived rewards and costs of engaging in a health-related activity are key determinants of the intention to change and maintain behavior. The present findings indicate that health providers should be aware that PA is a health-related behavior that demands an intertemporal decision or trade-off between long-term and short-term behavioral outcomes. For example, a participant who engages in regular PA may make an immediate investment of time or money to achieve long-term health rewards. Heavy thoughts about how behavior will affect the future increases the intention to perform PA and finally lead to the actual PA behavior (Murphy and Dockray, 2018). Thus, in the future, it is imperative to compare and analyze the utility of a future-oriented intervention, which makes people think more about the long-term benefits of PA, and a present-oriented intervention, which helps people forego immediate things that prevent them from engaging in healthy behavior (Murphy and Dockray, 2018).

Second, in line with prior research, the final model developed in this study revealed that intention was one of the strongest determinants of PA behavior. This reinforces the previous concepts that strong intentions are particularly important for initiating healthy behavior (Mullan and Novoradovskaya, 2018). Although habit strengths and self-regulatory strategies play significant roles in the maintenance of health-related behaviors over time (Gardner et al., 2020), it has been suggested that sustained intrinsic motivation or strong intentions are required for long-term behavioral maintenance (Phillips and Mullan, 2022). Therefore, while developing interventions to increase PA levels, people's unique characteristics and preferences should be considered (Novoradovskaya et al., 2020).

Third, health providers should adopt a more balanced view that also considers the automatic process of PA behavior apart from focusing on the motivational sphere of PA behavior. A recent randomized controlled study indicated that individuals who participated in an intervention aimed at habitually engaging in MVPA showed increased MVPA minutes per week after 8 weeks (Kaushal et al., 2017). It is, therefore, necessary for healthcare practitioners to monitor the occurrence of PA habits among patients with CHD. Habits may be formed by the use of clues and by establishing stable environments (Kaushal et al., 2018). A supportive environment may be necessary for patients with CHD to develop and maintain the habits of PA. A prospective cohort study of 846 patients who had coronary artery bypass (CABG) surgery found that those who resided in the greenest quarter of their neighborhoods had 52% higher odds of being physically active (Sadeh et al., 2021). Therefore, the government should create community sports facilities and venues that will provide favorable environmental conditions for the formation of PA habits among patients with CHD. In addition to environmental clues, monitoring and feedback mechanisms promote the maintenance of PA habits, which are mainly reflected through the achievement of PA goals, as well as the motivation from family members and peers (Aarts et al., 1997). Therefore, by evaluating the obstacles faced by patients in their efforts to become more physically active and utilizing evolving technology, such as accelerometers and smartphone applications, health providers can give technological assistance which can help individuals with monitoring, feedback, and motivation of PA behaviors (Sallis et al., 2015).

Finally, SC is necessary when one's short-term and long-term goals are at odds with one another (Tangney et al., 2004). It can help an individual to suppress immediate urges in favor of the more desirable long-term results (Hagger et al., 2010). Thus, healthcare practitioners should attempt to identify problematic SC aspects and anticipate events of self-regulation failure under specific circumstances, facilitators, and barriers (such as giving in to TV temptations). Interventionists should teach them a set of five self-regulation techniques (such as disregard, displace, distract, dispose, and diminish) to overcome automatic behaviors (Chew et al., 2021). Patients with CHD who can self-regulate can achieve the suggested amount of PA by the AHA/ACC guidelines by autonomously adopting self-regulation techniques to maintain planned behavior modification.

## Strengths and limitations

This study has several strengths. First, it integrates two evidence-based theories related to health behavior to investigate determinants of levels of PA. This is achieved by considering both rational and automatic determinants of PA. This is the first study to investigate the association between the TST variables and PA behavior in patients with CHD. Hence, our results are expected to stimulate further research and contribute to future theoretical and practical research. The positive relationship between CFC and intention to PA behavior suggests that interventions should focus on simulating the implications of current PA behaviors on their health and significant others in the future (Hollis-Hansen et al., 2019). A significant proportion of the variance in PA levels was accounted for by intention and habit, indicating that focusing on rational and/or automatic processes may promote PA levels. For instance, if the aim is to achieve a guideline-recommended level of PA, then interventions that raise motivation or establish this behavior as a habit might be effective. The interaction between intention and SC suggested that goal setting, action planning, and self-monitoring could be an effective combined intervention for turning intention into behavior (Chew et al., 2021).

Despite these findings, this study has some limitations that should be considered. First, our participants were recruited from a single clinical setting *via* convenience sampling; thus, our results may not apply to the wider population. However, by focusing on patients with CHD, the mechanics of achieving the guidelines-recommended level of PA were revealed in this study. In the future, studies should investigate the associations between the TPB and TST variables in various clinical settings. Similarly, in line with previous research based on the TST model (Tangney et al., 2004; Moran and Mullan, 2021), we measured CFC and trait SC, rather than specific to PA behavior. If specific PA-based measures of CFC and SC were used, greater variance in PA intention and actual behavior may have been accounted for by CFC and trait of SC. Therefore, future studies should use a combination of specific PA-based measurements to measure the variables of the TPB and TST models in the real world. Third, this prospective study was conducted for 1 week; thus, the results cannot explain the effects of the variables of the TPB and TST models on intentions to actual PA behavior in the long term. Future longitudinal studies to investigate long-term effects on the maintenance of PA behavior are advocated. Thus, this correlational investigation should be interpreted with caution. Given that this study is not based on experiments, we cannot draw strong conclusions regarding whether SC or habit strength is associated with an increase in PA levels. In addition, there might be some level of reporting and social biases because all metrics were self-reported rather than direct observation (Rosenman et al., 2011); hence, our results should be interpreted with caution. Future research should aim to reduce subjective bias by using more objective psychometric experiments to measure psychosocial variables and accelerators to measure PA.

## Conclusion

The moderated mediation model based on the integration of the TPB and TST models fits the data well, suggesting that both rational and automatic processes are involved in the generation of PA behaviors in patients with CHD. Nevertheless, not all hypotheses were supported by the data (e.g., SN did not relate to the intention to achieve recommended-level of PA, SC did not directly relate to behavior, the intention could not mediate the relationship between SN and PA levels, and habit strength did not moderate the relationship between intention and PA levels). Further research should be conducted to confirm and build upon these results using objective measurements.

## Data availability statement

The original contributions presented in the study are included in the article/Supplementary material, further inquiries can be directed to the corresponding author.

## Ethics statement

Written informed consent was obtained from the individual(s) for the publication of any potentially identifiable images or data included in this article.

## Author contributions

Conceptualization: WW, MW, and XZ. Data collection and methodology: WW, YH, and MW. Formal analyses: WW and MW. Writing—original draft: WW. Writing—review and editing: WW, MW, YH, XZ, and GF. All authors contributed to the article and approved the submitted version.
